# Probiotic vs. placebo and metformin: probiotic dietary intervention in polycystic ovary syndrome – A randomized controlled trial

**DOI:** 10.1186/s12902-023-01294-6

**Published:** 2023-04-17

**Authors:** Valentin Borzan, Regina Riedl, Barbara Obermayer-Pietsch

**Affiliations:** 1 Division of Endocrinology and Diabetology, Medical University of Graz, Graz, Austria; 2 Center for Medical Biomarker Research CBmed, Graz, Austria; 3 Institute for Medical Informatics, Statistics and Documentation, Medical University of Graz, Graz, Austria; 4 Department of Internal Medicine, Division of Endocrinology and Diabetology, Endocrinology Lab Platform, Medical University of Graz, Auenbruggerplatz 15, 8036 Graz, Austria

**Keywords:** PCOS, Probiotic, RCT, Metformin

## Abstract

**Background:**

Polycystic Ovary Syndrome (PCOS) is a very common endocrine disorder with a variety of symptoms. Current treatment options include the contraceptive pill as well as metformin, however both treatments are limited to specific symptoms and have common side effects.

**Methods:**

This phase IV study is a monocentric, double blinded randomized clinical trial comparing the effects of six months of probiotic intervention to a placebo, with an additional open-label metformin arm as a positive control in a total of 180 participants with PCOS. The first of three visits is the screening visit, where inclusion/exclusion criteria are assessed. At the first visit, they are randomised into one of the three treatment arms equally and receive their study medication. After six months, all assessments from the first two visits are repeated. The primary endpoint is the change in free testosterone levels after the intervention, while secondary endpoints include changes in hormonal and metabolic parameters associated with PCOS as well as the gut microbial composition and diversity after intervention.

**Discussion:**

Based on new insights into the role of the gut microbiome in PCOS development, this study is exploring the potential of using probiotics to treat women with PCOS symptoms. If successful, this new therapy approach could open a new realm of possibilities for treating PCOS. To our knowledge, this is the first study comparing probiotic intervention with not only placebo treatment, but also metformin. This study has been approved by the ethics committee of the Medical University of Graz (EC number 32–230 ex 19/20).

**Registration:**

EudraCT number: 2020-000228-20. Clinicaltrials.gov identifier: NCT04593459.

**Protocol version:**

Version 1.5 dated 29th November 2021.

## Background

### PCOS and current therapeutic options

Polycystic Ovary Syndrome (PCOS) is a hormonal and metabolic disorder affecting up to 20% of women worldwide, depending on the diagnostic criteria used [1]. A very common definition of PCOS based on the revised Rotterdam criteria includes at least two out of three symptoms [2]: oligo-/amenorrhea, polycystic ovarian morphology and clinical or biochemical hyperandrogenism. Several comorbidities are also associated with PCOS, including depression, obesity, insulin resistance and Hashimoto’s thyroiditis [3, 4].

As the exact pathomechanisms of PCOS have not yet been established, therapeutic options mainly focus on specific symptoms. For obesity, insulin resistance and other metabolic symptoms, lifestyle changes, weight loss and off-label use of metformin are most commonly recommended and prescribed. Combined oral contraceptives (COCs) are most frequently used to alleviate hormonal symptoms (oligo-/amenorrhea, hirsutism) [3]. However, both metformin and COCs have potential side effects and contraindications, thereby limiting their use for the affected patients [3, 5].

### PCOS and microbiome interactions

Our group was among the first to discover lower bacterial abundances and significant differences in stool microbiome composition between women with PCOS and healthy controls in 2017 [6]. Since then, several studies have been published confirming these findings [7, 8]. The decreased alpha diversity of the gut microbiome has been found to correlate to total testosterone levels as well as the degree of hirsutism [7], while also being linked with insulin resistance, dyslipidemia as well as obesity [9, 10].

Gut microbiota also play a critical role in phytoestrogen metabolism in our body. For women with PCOS, the isoflavone metabolite equol may be of particular interest. Daidzein, one of the most abundant isoflavones found in plant products, can only be metabolized to equol via certain bacterial species in the human gut [11]. Equol in turn has been shown to bind to the estrogen receptors alpha and beta and to have anti-androgenic, anti-cancer and anti-inflammatory effects [12–15].

Several studies have already tested probiotic products for therapeutic use for women with PCOS [16–22]. Most studies had exactly 60 participants with PCOS randomized to two groups, however recent RCTs found very promising results when using probiotics to treat PCOS symptoms, with several studies reporting a reduction in androgen levels as well as improving metabolic markers [23, 24]. A recent metaanalysis of RCTs conducted until 2019 with this topic in mind also found an improvement in metabolic and hormonal markers in women with PCOS across 11 analyzed studies [25], as did another review on the benefits of various nutritional approaches [26].

### Hypothesis and objectives

The working hypothesis of this randomized controlled trial (RCT) is an improvement of PCOS symptoms and androgen levels after six months of probiotic intervention. To that end, the trial is designed to primarily compare the probiotic intervention to a placebo treatment and secondarily to a standard non-hormonal treatment option such as metformin. To our knowledge, this RCT is the first trial to compare the effects of probiotics to placebo and to include metformin as a benchmark treatment arm.

Two other secondary objectives are to determine the prevalence and impact of equol production in women with PCOS compared to previous reports in literature based on the general population [27, 28]. The fourth secondary objective is to obtain biopsy samples from 30 voluntary participants across all three treatment arms from the stomach, the duodenum as well as the sigmoid colon in order to assess potential differences between the luminal and mucosal microbiome composition in the study subjects.

## Methods and analysis

### Study design

This phase IV trial is monocentric, randomized and double-blinded with one additional open-label treatment arm serving as the positive control. Two randomizations are performed. In the first randomization, participants are randomized in a 2:1 ratio to either the double-blind part or open-label metformin part. This open randomization is performed via the web-based randomization service “Randomizer” (Randomizer; Institute for Medical Informatics, Statistics and Documentation, Medical University of Graz, Austria; available at: www.randomizer.at, accessed: September 3, 2021).

The patients who are randomized to the double-blind part are further randomized in a 1:1 ratio to either the probiotic group or the placebo group. The second randomization as well as the allocation concealment is performed by Winclove Probiotics B.V, Netherlands. To ensure that the 30 participants undergoing endoscopic procedures are balanced over the three groups, both randomizations are stratified for endoscopy (yes/no). Both randomizations are performed at visit 1.The study takes place at Graz University Hospital, Department of Internal Medicine, Division of Endocrinology and Diabetology, Endocrinology Outpatient Clinic.

The treatment arms are the following:

1. Probiotic (number n = 60).
2. Probiotic placebo (n = 60).
3. Open-label metformin (500 mg twice daily, n = 60).

**Fig. 1:**
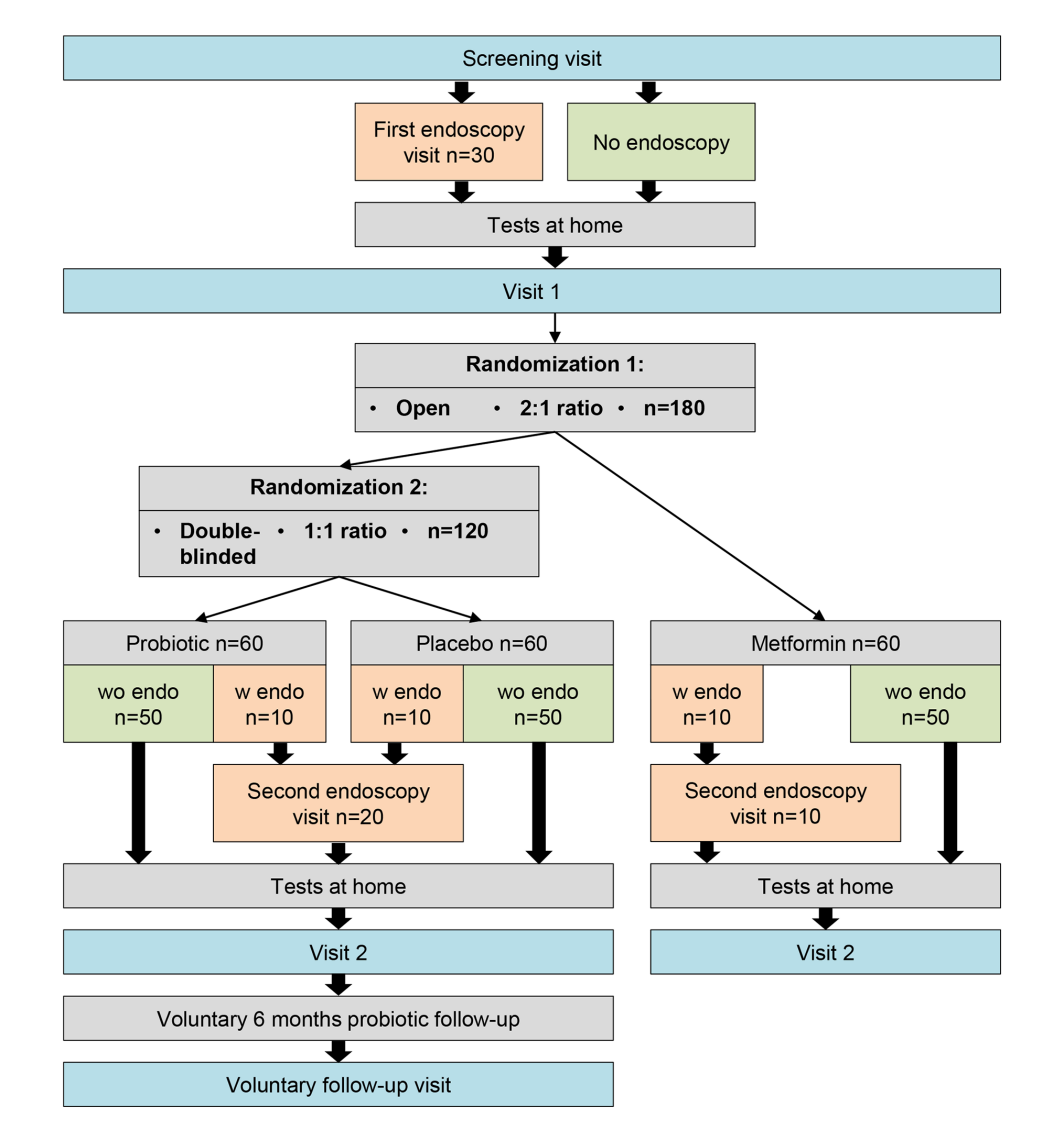
Study design of the ProPCO-RCT study; First, the participants´ inclusion/exclusion criteria are determined with detailed patient histories, questionnaires and blood analysis at the screening visit. Then, 30 out of the total 180 participants voluntarily submit to an endoscopy in order to obtain gut biopsy samples for detailed microbiome analysis. Next, all participants perform a soy challenge test and a gut permeability test at home and collect stool and urine samples, which they return at visit (1) There they will be randomized into either the open-label metformin arm or the double-blinded probiotic-placebo arms, with the 30 endoscopy participants being allocated equally across the three groups. If they are randomized into the probiotic or placebo groups, they receive a randomized package containing the blinded trial substances. They then take the study medication for 6 months, before repeating both the endoscopies and the tests at home and returning for visit (2) There all assessments from the screening visit are repeated. Participants of the probiotic and placebo arms may prolong their participation by taking the probiotic product in a 6-months follow-up period, before returning for the final follow-up visit, which is identical to the visit 2; w endo: with a scheduled endoscopy visit; wo endo: without a scheduled endoscopy visit;

The framework of the study is to show that the effects of the probiotic intervention are superior to the placebo arm. The metformin arm serves as a standard benchmark treatment arm and positive control. The study design is outlined in Fig. 1. This study protocol adheres to the SPIRIT recommendations.

### Timeline

For this purpose, potential participants have a screening visit, where participants give written informed consent, blood and urine samples are collected and extensive patient history as well as questionnaires on PCOS symptoms, probiotic intake, depression symptoms and diet are collected. A soy challenge test as well as a functional gut permeability test are scheduled at home, requiring the participants to take urine and stool samples. For the soy challenge test, participants consume 2 × 250 ml soy drinks for two consecutive days, followed by urine sample collection at home each morning the day after soy consumption, similar to Setchell et al. [29]. In the morning of the second urine collection day, participants then drink a sucrose-mannitol-lactulose solution followed by 5 h of urine collection to measure gut permeability. Stool samples are collected before and after soy consumption.

At the first visit (taking place four weeks at the latest after the screening visit), participants deliver the collected stool and urine samples and receive a randomized package of intervention product, which they will take for the next six months. The probiotic and placebo arms get 200 sachets each respectively, to be taken once daily. The metformin group receives 400 tablets of 500 mg metformin, to be taken once daily for the first 7 days of intervention before increasing the dosage to twice daily.

Participants are asked to document any significant health changes or dietary intakes as well as the first day of each menstrual cycle. The intervention is scheduled to last 161 to 180 consecutive days, depending on the date of the second visit.

At the end of their intervention, they will repeat the isoflavone challenge and the functional gut permeability tests at home and deliver the respective samples to the outpatient clinic at their second visit (taking place 24–26 weeks after the first visit). There, the blood and urine tests from the screening visit will be repeated.

Participants in the blinded arms of the trial have the option to further take part in a follow-up period of an additional six months, during which they would take the probiotic formula (without placebo), followed by a follow-up visit scheduled one year after trial enrollment.

In addition, two gastro-/sigmoidoscopies each will be performed at the beginning and the end of the intervention period in 30 voluntary participants (10 from each treatment arm planned) in order to obtain biopsy samples from the stomach, duodenum and sigmoid colon. These samples are used for the assessment of the mucosal gut microbiome as opposed to the luminal gut microbiome composition in stool samples. An overview of the timeline and visit schedule is provided in Table 1.

**Table 1:** Overview of number of visits and outcome assessments

Visits	Screening visit	Endoscopy 1	Visit 1	Endoscopy 2	Visit 2 End of Intervention	Voluntary follow-up
Weeks +/- 3 or more days	(-4)-(-1) (+/-3 days)	-1 (+/-3 days)	0 (+/-3 days)	24–26 (+/-3 days)	26 (+/-3 days)	52 (+/-3 days)
**Patient history**						
Extensive patient medical history	x					
Changes in medical history			x		x	x
PCOS history and symptoms	x				x	x
**Randomisation**			x			
**Endoscopic procedure**		x		x		
**Examinations**						
Body weight/height	x				x	x
Waist/Hip circumference	x				x	x
Blood pressure, heart rate	x				x	x
Ferriman-Gallwey Score	x				x	x
**Questionnaires**						
Diet (General, vitamin D and calcium)	x				x	x
PCOS	x				x	x
Depression scales	x				x	x
Bristol Stool Scale	x				x	x
**Laboratory**						
Hormone panel	x				x	x
Oral glucose tolerance test	x				x	x
Safety lab (not an endpoint)	x				x	x
Coagulation safety lab	x					
FACS	x				x	x
P800 and PAX gene tube	x				x	x
Inflammation, gut permeability	x				x	x
Urine tests	x				x	x
Pregnancy tests	x				x	x
Biobank samples	x				x	x
**Isoflavone intake test**	Between first and screening visit			Before the second visit		
**Functional gut permeability test**	Between first and screening visit, after isoflavone intake test			Before the second visit, after isoflavone intake test		

### Eligibility criteria

In order to be eligible for the trial, women should be between 18 and 45 years of age, they should have at least two out of three Rotterdam criteria and they have to sign the informed consent form before starting the trial.

Women can only participate if they do not have: hyperandrogenism of any cause other than PCOS; pregnancy or nursing period; soy or other allergies related to the study procedures; type 1 diabetes; chronic inflammatory bowel disease, history of cancer in the gastrointestinal tract or acute gastrointestinal infection; any malignancies requiring treatment in the 3 years preceding trial participation; any other chronic disease requiring medical check-ups or hospital treatments at least once every three months (exception: type 2 diabetes); major surgery in the gastrointestinal tract (e.g. colectomy, gut segment excision with stoma surgery, Whipple´s surgery; removal of the appendix or the gall bladder is not considered major surgery); chronic therapy with antidiabetic drugs, proton pump inhibitors, systemic intake of steroids or hormonal contraceptives in the six months preceding trial participation; antibiotic treatment within three months prior to trial procedures; alcohol or drug abuse.

### Intervention

#### Probiotic and placebo

The main intervention in this trial is a dietary food supplement powder containing seven probiotic bacterial strains: lactobacillus salivarius W57, lactobacillus casei W56, lactobacillus rhamnosus W71, lactococcus lactis W58, enterococcus faecium W54, lactobacillus plantarum W62 and lactobacillus acidophilus W22 (manufactured by Winclove Probiotics B.V., Netherlands; marketed as OMNi-BiOTiC® metabolic by Institut AllergoSan GmbH, Graz, Austria) In addition, the probiotic contains 2000 IU cholecalciferol. Other ingredients are corn starch, maltodextrin, fructo-oligosaccharides, galacto-oligosaccharides, polydextrose, plant proteins, potassium chloride, magnesium sulfate, manganese sulfate and lactose. By comparison, the placebo is very similar to the probiotic visually and in taste and smell. Its ingredients are corn starch, maltodextrin, potassium chloride, magnesium sulfate and manganese sulfate. Both Verum and Placebo are packaged in blank sachets, so that patients, caregivers, investigators and outcome assessors are blinded to their content.

On each sachet, the expiration date and the study acronym (ProPCO-RCT) is clearly shown. 50 sachets are packed in one box, where the instruction for ingestion, the expiration date, the randomization code and the ingredients are noted. The bacterial strains are mentioned on every box with the symbol +/- (regardless if the strains are included or not). Every participant receives 4 boxes, in total 200 sachets. An excess number of sachets (the intervention period is only 180 days) is included in case the participant loses a few.

#### Metformin

The metformin drug used in this trial is Glucophage, a commercially available extended release metformin-hydrochloride pill, owned by Merck GmbH, Vienna, Austria. It is packaged in boxes containing 200 pills with no special markings regarding the trial.

#### Blinding

The blinding is applied to the trial participants and the investigators, who serve as both care providers and outcome assessors.

A treatment code table was generated via Winclove Probiotics B.V., Netherlands. Each Verum/Placebo box containing 50 sachets receives a unique code, corresponding to the treatment code table. The blinding of the sachet boxes is also done at Winclove Probiotics B.V., Amsterdam, Netherlands.

To ensure proper stratification of the 20 individuals taking probiotics/placebos who also have two endoscopies during the trial, the randomisation is separated into two lists: The first list contains the 100 individuals without endoscopies, and the second list consists of the 20 participants that are assigned to the endoscopic procedures. The investigators have both lists available and assign participants the corresponding code in ascending order.

Since we are only randomizing dietary supplements and placebos in a blinded manner in healthy participants, we do not expect any complications from the trial participation that would require emergency unblinding. However, in case of a medical emergency involving a participant of the trial, study investigators may be reached through the Endocrinology Ward, Division of Endocrinology and Diabetology, Medical University of Graz on a 24/7 basis.

The randomization can be unblinded at the Endocrinology ward, where 120 sealed envelopes contain the allocation of each number. Only the envelope containing the treatment number for the patient in question may be opened. Both the principal investigator and the sponsor have to be notified as soon as possible of the emergency unblinding.

#### Concomitant therapies

Apart from the metformin and the probiotic formula as well as the placebo, no additional medicinal products will be administered by the investigators as part of this study. However, a subgroup of PCOS patients has additional diagnoses and therefore we need to account for any medications the participants might have to take. If a participant takes any non-permissible concomitant therapy (for example antibiotic treatment), this has to be documented in the CRF at the next visit. Thyroid hormone derivatives, renin-angiotensin-aldosterone system inhibitors (such as angiotensin-converting enzyme inhibitors, angiotensin I and II inhibitors), as well as topical or locally applied ointments, drops, creams, salves, gels and powders (provided their ingredients do not have a systemic effect) may be taken during the trial without further need for documentation.

In contrast, antibiotic treatments, metformin and other antidiabetic drugs, antacid medication (proton pump inhibitors), steroid hormone treatments as well as probiotic products (not including probiotics contained in food products such as yoghurt) should not be taken during the trial as well as prior to the trial based on the exclusion criteria. Should a situation require a trial participant to take any of these substances (such as an antibiotic due to infection), the circumstances and duration of the drug intake should be documented in the CRF at the next visit.

### Outcome parameters

#### Primary outcome

The primary endpoint of the study is a change in serum free testosterone concentration in women with PCOS after a 6-month intervention with probiotics compared to placebo treatment.

PCOS combines several different phenotypes and symptoms which may vary greatly, however free testosterone is one of the most consistent biomarkers of PCOS and its symptoms and therefore the most suited for designation as the primary outcome measure and may provide the most benefit if successfully lowered due to the intervention [30].

#### Secondary outcomes

- Glucose metabolism via HOMA and Matsuda indices as determined through an oral glucose tolerance test.
- Other hormonal parameters of PCOS (Anti-Müllerian hormone; androstenedione; follicle-stimulating hormone FSH; luteinizing hormone LH; dehydroepiandrosterone-sulphate DHEA-S; 17α-hydroxy-progesterone; 17β-hydroxy-estradiole; total testosterone, 25-hydroxy-cholecalciferol).
- Hirsutism (modified Ferriman-Gallwey score).
- Body weight (body-mass-index BMI; waist-to-hip-ratio).
- Gut permeability and inflammation (functional sucrose-lactulose-mannitol test; surrogate parameters: serum diaminooxydase; stool zonulin; calprotectin; lipopolysaccharide; soluble cluster of differentiation sCD14; bacterial DNA).
- Gut lumen and mucosa microbiome composition (16 S-RNA gene sequencing).
- Phytoestrogen production -log10 (equol to daidzein concentration ratio in urine) obtained via the soy challenge test)
- Quality of life (PCOS questionnaire; depression questionnaires; diet questionnaires).

#### Exploratory outcome measures

- Lipid metabolism (low-density lipoprotein LDL; high-density lipoprotein HDL; lipoprotein a LP(a); triacylglycerol;)
- FACS analysis (B cell subtypes).
- Metabolomics of stool and blood.
- Gene expression analysis in blood and biopsy samples.
- Changes in incretin levels.

### Statistical considerations

This study is conducted to compare two treatment groups with each other in respect to PCOS parameters. In the double-blind part, comparisons between the probiotic and the placebo arms will be performed to show that probiotics are superior to placebos. The comparison of the open-label metformin arm with the probiotic arm will be exploratory.

#### Sample size calculation

Prior to this RCT, we conducted a short pilot trial with 30 women with PCOS (EC number 30–205 ex 17/18), randomly assigning 10 women into three probiotic intervention arms respectively, both to test our methodology as well as to assess the feasibility of improving PCOS symptoms with probiotics (Borzan V., Sommer F., Riedl, R., Obermayer-Pietsch B., 2020, data on file).

In the pilot study 10 participants received OMNi-BiOTiC® metabolic for 3 months and showed a median (minimum-maximum) reduction in free testosterone levels of -0.3 pg/ml (-1.7-2.5). We assumed that over 6 months the reduction in the probiotic group would be improved to -0.6 pg/ml, which corresponds to 20% of the free testosterone levels found in healthy women based on the cut-off value of 3,16 pg/ml free testosterone in our Endocrinology Lab platform. A sample size of 54 in each group would have 80% power to detect a difference in means of -0.6 pg/ml assuming that the common standard deviation was 1.1 pg/ml using a two group t-test with a 5% two-sided significance level. Including a 10% dropout, 60 participants per group had to be included. For the metformin group in the open part of the trial, 60 participants were recruited as well. Therefore, the total anticipated sample size was 180.

#### Statistical analysis of outcomes

The analysis of the collected data in the study will be performed with SAS v9.4. The primary analysis will be performed on the intention-to-treat population. Details regarding the defined populations and the statistical analysis will be provided in the statistical analysis plan (SAP). The SAP will be prepared before unblinding the trial.

The analyses described below will be performed for the comparison of the probiotic and the placebo arms and the probiotic and the metformin arms, separately.

Demographic and baseline characteristics will be summarized and compared between the groups descriptively. The data will be presented as summary tables and, where appropriate, as plots. Continuous variables will be presented as means, standard deviation, median, minimum and maximum, for categorical data frequencies and relative frequencies will be used.

The primary outcome, changes in serum free testosterone concentration from baseline (screening visit) to 6 months (visit 2) will be compared between the probiotic and the placebo arms by analysis of covariance (ANCOVA) with serum free testosterone concentration at month 6 as dependent variable, and the baseline serum free testosterone concentration and treatment group as covariates. If the assumptions of the ANCOVA are not fulfilled, the changes in serum free testosterone concentration (i.e. difference visit 2 - baseline) will be compared between the groups by t-test or Mann-Whitney-U-test. A two-sided p-value of < 0.05 is considered to indicate statistical significance.

For the secondary and exploratory endpoints, group comparisons will be performed using parametric or nonparametric methods for unpaired data (as appropriate). I.e. the continuous secondary endpoints will be analysed similarly to the primary endpoint. In addition, linear mixed models will be used to analyse the group difference over the three time points (baseline, visit 2 and follow-up visit).

The collected stool samples will be sequenced with Illumina and the raw data will be uploaded on the Galaxy server of the Medical University of Graz. Microbiome analysis will be conducted using the QIIME 2 pipeline of the Galaxy server, the samples will be compared in respect to alpha- and beta-diversity using established methods. The results can be downloaded directly from the server.

### Adherence to study procedures

Due to stringent exclusion criteria and the long duration of the intervention period, we expect many participants to not be able to fully adhere to study procedures as outlined in the study protocol due to for example requiring antibiotic treatment or an unscheduled pregnancy. The study was planned and submitted prior to the COVID-19 pandemic outbreak, and therefore in many cases appointments and schedules for trial procedures may need to be altered. In such a case, deviations from the study protocol are documented in the CRF and participants may still continue with trial participantion. For the purposes of statistical analysis, all participants will be considered for the intention-to-treat analyses. The same statistical analyses will be applied additionally for the subpopulation that completely adhered to the trial protocol.

### Responsibilities

This is an investigator-initiated trial, with Barbara Obermayer-Pietsch, Prof. MD acting as the principal investigator as well as the sponsor in the name of the Medical University of Graz, thereby having all rights and responsibilities of both roles. Additional investigators are Valentin Borzan, MD, Stefan Pilz, MD, PhD, Christian Trummer, MD, PhD, Verena Theiler-Schwetz, MD, PD, Marlene Pandis, MD and Claudia Stiegler, MD. Roswitha Gumpold is the study nurse involved in the trial, and Regina Riedl, PhD is the biostatistician responsible for statistical analysis. Harald Kojzar, an independent data monitor, is assigned by the sponsor of the trial to oversee and support trial procedures and personnel respectively.

### Data collection and handling

For data collection, the newest version of SPSS (currently Version 26) will be used.

The results from the medical history and the physical examination are entered in the CRF. In addition, we will document the visit progress and whether every test and procedure was completed according to the protocol. This includes the laboratory codes used to identify the lab results, the intervention code from the sachets (or the allocation to metformin), as well as any adverse events.

For monitoring purposes, data from the paper CRF will be entered into an online CRF available at the Phoenix database of the Medical University of Graz.

The lab results from the study will be measured at the Endocrinology Lab Platform and the Clinical Institute of Medical and Chemical Laboratory Diagnostics and entered into the hospital patient information program “MEDOCS”, with the study-specific code attached to the data. From there, all results from the study can be downloaded in an excel sheet using the study code. Only authorized personnel have access to MEDOCS and only study-related personnel may access the study-specific results. All collected data will then be transferred to SAS for further analysis.

### Storage and data protection

This study adheres to legal regulations for the storage, registration, transfer and evaluation of data according to the Austrian Act on Pharmaceutical Products and data protection law (AMG). By signing the consent form, potential participants are informed and agree to the following:

Data obtained during the course of this clinical trial will be treated as strictly confidential, and passed on exclusively to the following persons without mentioning names (pseudonymised): the sponsor of the trial for scientific evaluation and assessment of adverse events as well as the biostatistician responsible for outcome assessment. Investigators responsible for the recruitment and data collection during the trial as well as the lab personnel responsible for outcome parameter measurements may work with personalized data (not pseudonymized) when required.

Monitoring and auditing personnel, employees of national and foreign health authorities as well as members of the competent ethics committee may also gain access to personal data at the study centre for auditing and quality control purposes.

The participant is entitled to terminate her participation in the clinical trial at any time without stating reasons and without future consequences or disadvantages. However, we are required by law to store and in individual cases inspect personal data even after trial dropout for a period of time as defined in the AMG.

### Data dissemination and publication

Any data obtained via this RCT may be published in conference papers, talks or posters, as well as peer-reviewed journals. This RCT is conducted as part of the PhD thesis of Valentin Borzan, MD and any data, figures and publications from this RCT may be used and cited in the doctoral thesis by Valentin Borzan, MD.

Should any of the results from this study be considered for publication, the publication guidelines from the Medical University of Graz and the GCP guidelines must be followed. The decision to publish the results of the trial is made by Barbara Obermayer-Pietsch, Prof. MD. as the principal investigator and sponsor of the trial. Prior to publication, the respective materials are sent to the area leader at CBmed as well as a representative of Institut AllergoSan for approval and acknowledgment respectively.

## Data Availability

The datasets used and/or analysed during the current study are available from the corresponding author on reasonable request.
